# Strategies to improve the implementation of healthy eating, physical activity and obesity prevention policies, practices or programmes within childcare service

**DOI:** 10.1017/S1463423621000219

**Published:** 2021-04-22

**Authors:** Daksha Trivedi

**Affiliations:** Senior Research Fellow, Centre for Research in Public Health and Community Care, University of Hertfordshire, Hatfield, UK

**Keywords:** child services, dissemination, health behaviour, implementation programme, research translation

## Review question

### Primary

To test the effectiveness of strategies aimed at improving the implementation of policies, practices or programmes by childcare services that promote child healthy eating, physical activity and/or obesity prevention.

### Secondary

To examine the effect of strategies on:

- the cost or cost-effectiveness
- adverse outcomes
- diet, physical activity or weight in children
- the acceptability, adoption, diffusion (penetration), sustainability and appropriateness of implementation strategies.

### Relevance to primary care and nursing

Primary health care professionals including specialist trained children’s nurses have a pivotal role in supporting children and families with physical activity (National Institute for Health and Care Excellence (NICE), 2009), nutrition (NICE, 2014) and preventing obesity (NICE, 2015). As such, understanding how these programmes are delivered in other settings that provide care to children can help with tailoring such support.

### Characteristics of the evidence

This Cochrane review contains 16 randomised controlled trials (RCTs) and 5 nonrandomised studies involving 1945 childcare services including preschools, nurseries and long day care services for children aged up to 6 years (Wolfenden *et al.*, 2020). They were conducted in the United States (*n* = 12), Australia (*n* = 8) and Ireland (*n* = 1). Studies examined various strategies to improve the implementation of any healthy eating, physical activity or obesity prevention policy, practice or programme in centre-based childcare services compared with no intervention, ‘usual’ practice or minimal support control (*n* = 19) or alternative intervention (*n* = 2). They were mainly delivered by trained public health practitioners and/or childcare educators.

### Summary of key evidence

Follow-up ranged from six months to two years. Pooled evidence ranged from low certainty to moderate certainty judged using GRADE (The Grading of Recommendations Assessment, Development and Evaluation), with a risk of bias, which was generally unclear for most aspects of the studies and high for dietary intake outcomes.

#### Primary outcome

Any measure of either the completeness or the quality of the implementation of childcare service policies, practices or programmes. This could include the proportion of services implementing foods in line with dietary guidelines and the number of compliant services.

#### Secondary outcomes

Costs or cost-effectiveness; adverse outcomes; diet, physical activity or weight in children; the acceptability, penetration, diffusion, sustainability and appropriateness of implementation strategies. Number of studies and number of participants (*n*) are given in parenthesis. Only significant effect sizes from meta-analysis of pooled studies are indicated below. They are presented as risk ratio (odds ratio, OR) for binary outcomes and a standardised mean difference (SMD) for continuous items, with 95% confidence interval (CI).

### Implementation strategy (educational materials/talks, audit and feedback, opinion leaders, small incentives or grants, educational outreach visits or academic detailing, reminders and tailored interventions)

Evidence from nine RCTs suggested an improvement in the implementation of programmes promoting child healthy eating, physical activity and/or obesity (SMD 0.49; 95% CI 0.19, 0.79; *n* = 495, moderate certainty evidence) and in the proportion of services or staff implementing a policy or practice (OR 1.83, 95% CI 0.81, 4.11; participants = 391 childcare services; low-certainty evidence).

There was no evidence of significant effect on adverse events from three studies of low-certainty evidence. No studies examined the cost or cost-effectiveness of strategies. There was no significant effect of interventions to improve implementation on diet, physical activity or weight status from a small number of studies.

Acceptability of implementation strategies was reported to be high in eight studies, ranging from 60 to 100%.

Penetration or diffusion of educational materials across 12 studies ranged from 37 to 100%. None of the included studies reported on adoption, sustainability or appropriateness.

### Implications for practice

Whilst research suggests that broadly implementation strategies can have a positive impact on the implementation of programmes, the evidence base is limited to guide policy and practice. Formative work for practitioners to understand the setting, context and barriers to implementation is important to enable successful implementation.

### Implications for research

Larger high-quality studies are needed to better understand the impact of these strategies. In addition, there is a need to measure cost-effectiveness and adverse effects. Implementation strategies informed by theoretical frameworks are needed to adequately address influencing factors to implementation. Future research should employ hybrid designs that measure both implementation outcomes and impacts on health behaviours.

## References

[ref1] National Institute for Health and Care Excellence (2009) Physical activity for children and young people. Retrieved 9 February 2021 from https://www.nice.org.uk/Guidance/PH17

[ref2] National Institute for Health and Care Excellence (2014) Maternal and child nutrition. Retrieved 9 February 2021 from https://www.nice.org.uk/Guidance/PH11

[ref3] National Institute for Health and Care Excellence (2015) Obesity in children and young people: prevention and lifestyle weight management programmes. Retrieved 9 February 2021 from https://www.nice.org.uk/guidance/QS94

[ref4] Wolfenden L , Barnes C , Jones J , Finch M , Wyse RJ , Kingsland M , Tzelepis F , Grady A , Hodder RK , Booth D and Yoong SL (2020) Strategies to improve the implementation of healthy eating, physical activity and obesity prevention policies, practices or programmes within childcare services. Cochrane Database of Systematic Reviews, Issue 2, Art. No. CD011779. 10.1002/14651858.CD011779.pub3. PMC7008062 32036618

